# On the Local Treatment of Pulmonary Cavities

**Published:** 1874-07-15

**Authors:** F. Mosler

**Affiliations:** Griefswald


### ON THE LOCAL TREATMENT OF PULMONARY CAVITIES.

By Prof. F. Mosler, of Greifswald. A Resume of the Author's Various Reports by the Editor of the Allgemeine Wiener Med. Zeitung.

*Translated for* The Examiner *by* Dr. H. Gradle. *Continuation from June 1st, No. XI.*

THE exuding pus became constantly less in quantity, and of a more laudable nature; whence Mosler concludes that this method of disinfection is of greater efficacy than inhalation of carbolic acid per orem. By percussion the cracked-pot resonance was now more distinctly perceived over the cavity, probably on occount of its external opening; the mucous rales, however, were less marked. The patient made no complaints about trouble with his lungs. The subjective condition improved after the operation, while the destruction of the lung seemed to have been arrested. An improvement, however, of the general health was not observed, nor, indeed, expected. The albuminuria increased constantly, the constitution being accordingly weakened, so that finally the patient could not leave his bed.

This comprises the reports which Mosler made at the last congress of naturalists at Wiesbaden; his deductions we will refer to, later.

When Mosler returned to Greifswald on the 1st of October, 1873, he found—as he reports in the *Berl. Klin. Wochenschrift*—a change for the worse, as the debility had increased in spite of tonics and stimulants, while albumen appeared in still greater quantities in the urine. Emaciation was very marked; appetite and bowels were normal; pyrexia was not present. The temperature was neither in the morning nor evening higher than 37.6° C. (99.6° F.); the cough had diminished, the sputa also, while the pus flowed still freely through the canula, on account of which inhalations of a diluted solution of carbolic acid were continued twice daily. The physical examination showed no great change; the destruction had evidently been arrested. Respiration had been but little disturbed so far, but occasionally there was a sense of fullness in the epigastrium. A notable exarcerbation set in on October 3d; cardiac activity became enfeebled, and signs of collapse appeared; the temperature sank to 36.6° C. (97.8° F.), and dyspnœa occurred. On the evening of October 5th death from cardiac paralysis closed the scene.

The autopsy revealed the following condition: The left lung well retracted; the pleural layers of the right side completely adherent to each other; the left lung adherent at the apex, less along the posterior part of the lower lobe. On section, both the upper and lower lobes were shown to contain air; in their substance grayish-white nodules, partly discrete, partly arranged in circular groups, were found in small number. The mucous lining of the bronchial tubes was not altered. The right lung was adherent to the costal pleura in its whole extent, especially above the third rib. On removing the costal pleura, there was found, from the apex to the base of the upper lobe, a whitish pseudo-membrane of almost cartilaginous consistency, several lines in thickness. On the lower anterior part of the upper lobe, a canal with smooth edges was noticed, running in an upward antero-posterior direction (corresponding to the site of the canula) which led to a cavity occupying the greater part of the upper lobe, and filled with a yellow, creamy fluid. The interior of the cavity was traversed by septa of a reddish hue, contrasting with the dark gray color of the smooth walls. On some partitions the surface appeared feebly granular. From a section of the lower lobe there exuded on pressure a reddish watery fluid. Here also a small number of gray nodules, of the size of a millet seed, were seen, arranged as before stated. The heart was not much altered, its muscular tissue feeble and of a brownish color. The spleen and both kidneys showed distinct amyloid degeneration, likewise the villi on the interior of the ileum and jejunum; in some places the mucous membrane of these organs was denuded.

From the statements made at the congress of naturalists at Wiesbaden, Mosler concludes that the local treatment of pulmonary cavities is a practicable procedure.

The first suggestion in this line was made by Barry in the year 1726, and again thrown out subsequently by Nasse, Von Herff, and Hooker, without leading to a trial of the operation, since objections were constantly raised on account of the difficulty of a diagnosis and the obstacles to the performance of the operation; the immense progress, however, of medicine and surgery in the present time has overcome these obstacles. As regards the ultimate success and permanent value of the operation, views will differ, but a tolerance of modifications of the method can and ought to be expected. The above cases have at least proven that even as a symptomatic remedy the method is not without advantage. Whether the local treatment induces granulation, and consequent obliteration of the cavity, Mosler does not dare to decide before further observations have been made. At least this has been proven by his experiments, that the lung is much more tolerant of operative procedures, and that these are much less dangerous and difficult, than was formerly supposed.

As further proof of their innocuity, Mosler can state that since even limited pleuritic exudations are removed by aspiration in his clinic, it has happened occasionally that the trocar entered a pulmonary infiltration, instead of a pleuritic exudation, without injurious consequences to the patient. Similar observations were also communicated to the author by other physicians. Possibly we may even have the courage, at some future time, to treat pulmonary infiltrations, as other parenchymatous tumors, by injections of medicines.

